# Clinical and molecular characterization of SLC31A1-related developmental and epileptic encephalopathy: insights from 13 new cases

**DOI:** 10.1093/braincomms/fcaf348

**Published:** 2025-09-23

**Authors:** Natalia Juliá-Palacios, Gerard Muñoz-Pujol, Reza Maroofian, Aida M Bertoli-Avella, Marta Gómez-Chiari, Jordi Muchart-López, Abraham J Paredes-Fuentes, Mar O’Callaghan, Irene S Machado-Casas, Ingrid Cristian, Jennifer Morrison, Angels Garcia-Cazorla, Anna Codina, Mohammad Miryounesi, Emir Zonic, Peter Bauer, Huma Cheema, Muhammad Nadeem Anjum, Nouriya Al-Sannaa, Marwa Abd Elmaksoud, Faroug Ababneh, Sahar Alijanpour, Seyed Hassan Tonekaboni, Afshin Fayazi, Maria Urbaniak, Uxía Barba, Janet Hoenicka, Francesc Palau, Henry Houlden, Juan Darío Ortigoza-Escobar, Antonia Ribes, Carlos Santos-Ocaña, Millie Tyler, Patrick Gaffney, Christopher J Carroll, Frederic Tort, Klaas J Wierenga, Bryn D Webb, Rafael Artuch, Heidy Baide-Mairena, Roser Urreizti

**Affiliations:** Department of Pediatric Neurology, Hospital Sant Joan de Déu, IRSJD, 08950 Barcelona, Spain; Biochemistry and Molecular Genetics, Division of Inborn Errors of Metabolism-IBC, Hospital Clínic de Barcelona, 08028 Barcelona, Spain; Centro de Investigación Biomédica en Red de Enfermedades Raras (CIBERER), Instituto de Salud Carlos III (ISCIII), 28029 Madrid, Spain; Institut d'Investigacions Biomèdiques August Pi i Sunyer (IDIBAPS), 08036 Barcelona, Spain; Department of Neuromuscular Disorders, UCL Queen Square Institute of Neurology, WC1N 3GB London, United Kingdom; Medical Genetics and Reporting, CENTOGENE GmbH, 18055 Rostock, Germany; Department of Diagnostic Imaging, Hospital Sant Joan de Déu, IRSJD, 08950 Barcelona, Spain; Department of Diagnostic Imaging, Hospital Sant Joan de Déu, IRSJD, 08950 Barcelona, Spain; Biochemistry and Molecular Genetics, Division of Inborn Errors of Metabolism-IBC, Hospital Clínic de Barcelona, 08028 Barcelona, Spain; Institut d'Investigacions Biomèdiques August Pi i Sunyer (IDIBAPS), 08036 Barcelona, Spain; Department of Clinical Biochemistry, Hospital Sant Joan de Déu, IRSJD, 08950 Barcelona, Spain; Department of Pediatric Neurology, Hospital Sant Joan de Déu, IRSJD, 08950 Barcelona, Spain; Centro de Investigación Biomédica en Red de Enfermedades Raras (CIBERER), Instituto de Salud Carlos III (ISCIII), 28029 Madrid, Spain; Neuropediatrics and Neurodevelopment Unit, Hospital Universitario Clínico San Cecilio, 18007 Granada, Spain; Division of Genetics, Arnold Palmer Hospital for Children, 32806 Orlando, FL, USA; Division of Genetics, Arnold Palmer Hospital for Children, 32806 Orlando, FL, USA; Department of Pediatric Neurology, Hospital Sant Joan de Déu, IRSJD, 08950 Barcelona, Spain; Centro de Investigación Biomédica en Red de Enfermedades Raras (CIBERER), Instituto de Salud Carlos III (ISCIII), 28029 Madrid, Spain; Neurometabolism and Synaptic Metabolism Lab, Department of Neurology and MetabERN, Hospital Sant Joan de Deu, 08950 Barcelona, Spain; Applied Research in Neuromuscular Diseases, Hospital Sant Joan de Déu, IRSJD, 08950 Barcelona, Spain; Department of Medical Genetics, Faculty of Medicine, Shahid Beheshti University of Medical Sciences, 1985717413 Tehran, Iran; Medical Genetics and Reporting, CENTOGENE GmbH, 18055 Rostock, Germany; Medical Genetics and Reporting, CENTOGENE GmbH, 18055 Rostock, Germany; Pediatric Department of Gastroenterology, Children's Hospital of Lahore, 54000 Lahore, Pakistan; Pediatric Department of Gastroenterology, Children's Hospital of Lahore, 54000 Lahore, Pakistan; Johns Hopkins Aramco Health Care, 34465 Dhahran, Saudi Arabia; Department of Pediatrics, Neurology Unit, Faculty of Medicine, Alexandria University, 21526 Alexandria, Egypt; Department of Genetics and Precision Medicine, King Abdullah Specialized Children Hospital, King Abdulaziz Medical City, MNGHA, 11481 Riyadh, Saudi Arabia; King Abdullah International Medical Research Center, King Saud Bin Abdulaziz University for Health Sciences, 11481 Riyadh, Saudi Arabia; Department of Genetics, Faculty of Medicine, Alborz University of Medical Sciences, 3149969415 Karaj, Iran; Department of Pediatric Neurology, School of Medicine, Mofid Children's Hospital, Shahid Beheshti University of Medical Sciences, 15468 Tehran, Iran; Department of Pediatrics, School of Medicine, Hamadan University of Medical Sciences, 6517838678 Hamadan, Iran; Department of Clinical Biochemistry, Hospital Sant Joan de Déu, IRSJD, 08950 Barcelona, Spain; Department of Clinical Biochemistry, Hospital Sant Joan de Déu, IRSJD, 08950 Barcelona, Spain; Centro de Investigación Biomédica en Red de Enfermedades Raras (CIBERER), Instituto de Salud Carlos III (ISCIII), 28029 Madrid, Spain; Laboratory of Neurogenetics and Molecular Medicine, Center for Genomic Sciences in Medicine, Hospital Sant Joan de Déu, IRSJD, 08950 Barcelona, Spain; Centro de Investigación Biomédica en Red de Enfermedades Raras (CIBERER), Instituto de Salud Carlos III (ISCIII), 28029 Madrid, Spain; Laboratory of Neurogenetics and Molecular Medicine, Center for Genomic Sciences in Medicine, Hospital Sant Joan de Déu, IRSJD, 08950 Barcelona, Spain; Department of Neuromuscular Disorders, UCL Queen Square Institute of Neurology, WC1N 3GB London, United Kingdom; Department of Pediatric Neurology, Hospital Sant Joan de Déu, IRSJD, 08950 Barcelona, Spain; Centro de Investigación Biomédica en Red de Enfermedades Raras (CIBERER), Instituto de Salud Carlos III (ISCIII), 28029 Madrid, Spain; Biochemistry and Molecular Genetics, Division of Inborn Errors of Metabolism-IBC, Hospital Clínic de Barcelona, 08028 Barcelona, Spain; Centro de Investigación Biomédica en Red de Enfermedades Raras (CIBERER), Instituto de Salud Carlos III (ISCIII), 28029 Madrid, Spain; Institut d'Investigacions Biomèdiques August Pi i Sunyer (IDIBAPS), 08036 Barcelona, Spain; Centro de Investigación Biomédica en Red de Enfermedades Raras (CIBERER), Instituto de Salud Carlos III (ISCIII), 28029 Madrid, Spain; Centro Andaluz de Biología del Desarrollo, Universidad Pablo de Olavide, 41013 Sevilla, Spain; Cardiovascular and Genomics Research Institute, City St. George's, University of London, London SW17 0RE, United Kingdom; Genes and Human Disease Research Program, Oklahoma Medical Research Foundation, Oklahoma City, OK 73104, USA; Cardiovascular and Genomics Research Institute, City St. George's, University of London, London SW17 0RE, United Kingdom; Biochemistry and Molecular Genetics, Division of Inborn Errors of Metabolism-IBC, Hospital Clínic de Barcelona, 08028 Barcelona, Spain; Centro de Investigación Biomédica en Red de Enfermedades Raras (CIBERER), Instituto de Salud Carlos III (ISCIII), 28029 Madrid, Spain; Institut d'Investigacions Biomèdiques August Pi i Sunyer (IDIBAPS), 08036 Barcelona, Spain; Department of Pediatrics, Division of Genetics and Metabolism, University of Florida, Gainesville, FL 32610, USA; Department of Pediatrics, University of Wisconsin School of Medicine and Public Health, Madison, WI 53792, USA; Department of Genetics and Genomic Sciences, Icahn School of Medicine at Mount Sinai, New York, NY 10029, USA; Centro de Investigación Biomédica en Red de Enfermedades Raras (CIBERER), Instituto de Salud Carlos III (ISCIII), 28029 Madrid, Spain; Department of Clinical Biochemistry, Hospital Sant Joan de Déu, IRSJD, 08950 Barcelona, Spain; Department of Pediatric Neurology, Hospital Sant Joan de Déu, IRSJD, 08950 Barcelona, Spain; Department of Pediatrics, Hospital General Granollers, 08402 Barcelona, Spain; Centro de Investigación Biomédica en Red de Enfermedades Raras (CIBERER), Instituto de Salud Carlos III (ISCIII), 28029 Madrid, Spain; Department of Clinical Biochemistry, Hospital Sant Joan de Déu, IRSJD, 08950 Barcelona, Spain

**Keywords:** clinical delineation, CTR1 modelling, functional validation, brain MRI

## Abstract

Copper is indispensable for various metabolic processes, notably mitochondrial respiration. In humans, copper homeostasis hinges on transporters such as copper transporter 1 (CTR1), encoded by the *SLC31A1* gene. Recently, bi-allelic mutations in *SLC31A1* have been associated with a new neurodevelopmental disorder. This study presents clinical, genetic, and biochemical findings from 13 new cases across 10 families worldwide. RNA sequencing evaluated gene expression, and Western blotting assessed copper transporter 1 protein levels. Additionally, mitochondrial respiratory capacity was measured via high-resolution respirometry. Affected individuals exhibited a distinct clinical phenotype characterized by early-onset epileptic encephalopathy, severe neurodevelopmental delay and hypotonia, with high mortality. Neuroimaging revealed significant brain atrophy and white matter abnormalities. Genetic analysis identified bi-allelic *SLC31A1* variants, predominantly p.His120Gln in six cases and p.(Arg102Cys/His) in three cases. Functional studies in patient fibroblasts demonstrated impaired mitochondrial respiration. This study significantly broadens the clinical spectrum of this recently described syndrome, presenting as a severe developmental encephalopathy with high mortality risk, and suggests mitochondrial dysfunction as a potential pathomechanism. These findings contribute to the mounting evidence linking copper transporter 1 dysfunction to neurodegeneration, underscoring the urgency for further therapeutic investigations.

## Teaser

Juliá-Palacios, Muñoz-Pujol *et al*. reported 13 cases with bi-allelic *SLC31A1* mutations, presenting with early-onset epileptic encephalopathy, severe neurodevelopmental delay, and hypotonia, with high mortality. Preliminary functional studies showed impaired mitochondrial function in patient fibroblasts. These findings expand the clinical spectrum of *SLC31A1*-related disorder and suggest mitochondrial dysfunction as a possible underlying mechanism.

## Graphical Abstract

**Graphical Abstract:**
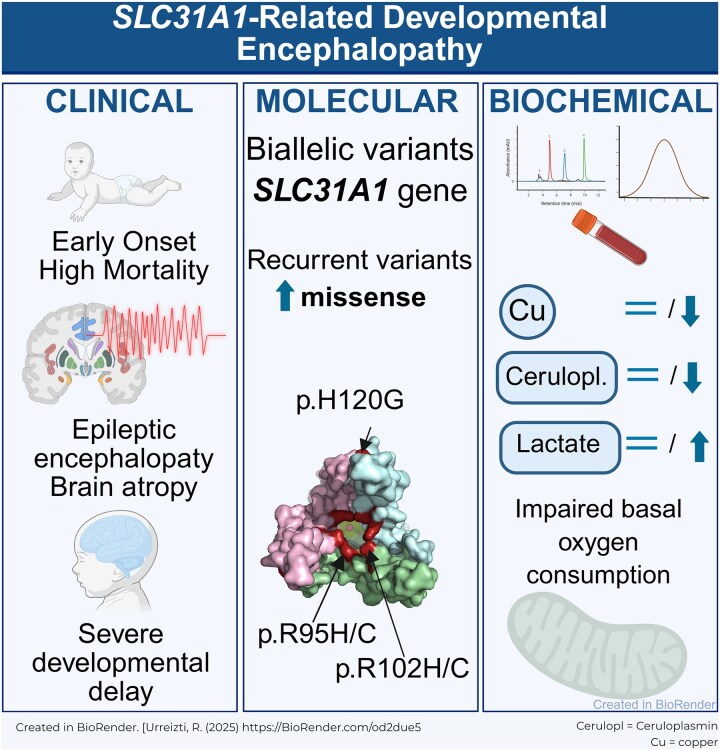

## Introduction

Copper (Cu) is an essential metal that is required for the proper functioning of metabolic pathways, including the synthesis of collagen and catecholamines. It is also the cofactor of cytochrome c oxidase in the mitochondrial respiratory chain, and of the antioxidant enzyme superoxide dismutase, among others. Thus, copper plays an important role in cellular respiration, antioxidant defence, connective tissue formation, neurotransmitter biosynthesis, neuropeptide amidation and iron homeostasis.^1,2^ There is evidence of copper homeostasis contribution in developmental and reparative angiogenesis.^3^ Intracellular Cu concentration is tightly regulated by dedicated proteins that facilitate its uptake, efflux, and distribution to target Cu-dependent proteins and enzymes.^4^ Two families of membrane proteins are critical for Cu homeostasis in mammals: the P-type ATPase copper pumps, ATP7A and ATP7B (MIM *300011 and *606882), that move Cu against a concentration gradient during Cu secretion or intracellular sequestration, and copper transporters (CTR) that are responsible for the initial uptake of Cu into cells. *AP1S1* (MIM *603531) encodes the small subunit σ1A of the adaptor protein-1 (AP1) complex, necessary for the intracellular trafficking of ATP7A. Through its involvement in clathrin-coated vesicle assembly, it directs the intracellular trafficking of copper pumps ATP7A and ATP7B.^5^ In humans, two CTR transporters have been identified, CTR1 (O15431) and CTR2 (O15432), encoded by the *SLC31A1* and *SLC31A2* genes, respectively (MIM *603085 and *603088). CTR1 is 190 amino acids long and forms a homotrimeric transmembrane transporter that is ubiquitously expressed, with relatively high levels in liver, small intestine, and brain (Lee *et al.*^6^ and GTEx Portal accessed August 2024). CTR1 is an endothelial Cu importer and functions as a redox sensor to promote angiogenesis in endothelial cells, as it has been demonstrated that oxidation of CTR1 at Cys189 promotes vascular endothelial growth factor receptor type 2 (VEGFR2) internalization and signalling to enhance angiogenesis in mice.^3^

Hitherto, different genetic disorders have been reported regarding Cu homeostasis. These include well-known Menkes disease (MIM #309400) and Wilson disease (MIM #277900), caused by pathogenic variants at *ATP7A* and *ATP7B* genes respectively, aceruloplasminaemia (MIM #604290) due to mutations in *CP* (MIM *117700), MEDNIK syndrome (MIM # 609313), a multisystemic disease that combines clinical and biochemical signs of both Menkes and Wilson’s diseases related to genetic defects in *AP1S1*, MEDNIK-like syndrome (MIM #242150) caused by mutations in *AP1B1* (MIM *600157),^7^ Huppke–Brendel syndrome (MIM #614482) associated with a severe loss of function of the endoplasmic reticulum (ER) membrane acetyl-CoA transporter 1 (AT-1) protein, encoded by *SLC33A1* (MIM *603690),^8^ and the very recently identified NSCT (neurodegeneration and seizures due to copper transport defect, MIM #620306) caused by recessive mutations in *SLC31A1*. Pathogenic variants in this gene have been reported in three cases with a deficiency in the high-affinity copper uptake protein 1 (COPT1; CTR1; O15431).^9,10^ This newly described genetic disorder leads to neurodegeneration and seizures and presents in the neonatal period or early infancy. Evidence of impaired intracellular Cu homeostasis was reported with intense expression of CTR1 in the basolateral aspect of the polarized choroid plexus epithelium, which mediates Cu uptake in the brain.^9^

Here we present thirteen new cases from ten different families. Among them, five cases from four unrelated families are homozygous for the same novel pathogenic variant in *SLC31A1* (NM_001859.4:c.360C > G; p.His120Gln). Through our comprehensive analysis, we describe the phenotype of the SLC31A1 deficiency-associated syndrome. Additionally, biochemical and molecular studies were conducted to further elucidate the underlying mechanisms of this condition.

## Materials and methods

### Subjects

Patients were recruited at different hospitals and genetic testing laboratory worldwide, and were identified through GeneMatcher platform.^11^ Demographic and clinical data (biochemical analysis, neuroimaging acquisition and neurophysiological studies) were collected by each attending physician by retrospective review of clinical charts and registered on a standardized form. Brain Magnetic Resonance Images (MRI) were acquired as part of the routine clinical assessment in each case, and five studies were systematically reviewed by an expert paediatric neuroradiologist (M.C.) to detail localization and characteristics of the lesions when present. Brain MRI reports were available for the rest of the seven cases.

### Ethical issues

This study was approved by the ethical committees of each institution and is in accordance with the Helsinki Declaration of 1964, as revised in 2001. The study was approved by the Hospital Sant Joan de Deu ethics committee with the reference no PIC-97-16.s. Family 4 was enrolled in a research protocol approved by the Institutional Review Board at the Icahn School of Medicine at Mount Sinai, Family 5 was enrolled in the Solve-RD—solving the unsolved rare diseases programme by the European Commission—and Family 6 was part of the URDCat programme. Adult participants and guardians of children provided written informed consent for participation.

### Laboratory methods

Biochemical analyses in blood, urine, and cerebrospinal fluid (CSF) were performed as part of routine diagnostic assessment at each centre. In **P7**, the analysis of lactate, pyruvate, amino acids, and organic acids was conducted using automated spectrophotometric procedures, UPLC-MS/MS and GC-MS/MS. The mitochondrial biomarkers FGF-21 and GDF-15 were analysed in serum by ELISA as described by Dominguez-Gonzalez *et al*.^12^ Copper measurements in serum, CSF, and fibroblasts were performed by ICP-MS, as previously reported.^13^

### Genomics and bioinformatics

All patients were diagnosed by next-generation sequencing (NGS) at their institutions according to their established pipelines (more information is available on demand). Contact among different institutions was done through the GeneMatcher platform^11^ and network of collaborators.

### mRNA expression analysis

mRNASeq was performed on **P7** fibroblasts. Quality control, library preparation, sequencing platform, and primary data analysis were done as previously described.^14^ Analysis of the aligned data was done using DROP (Detection of RNAseq Outliers Pipeline) in order to detect aberrantly expressed genes, altered splicing events, and monoallelic expression (MAE) of rare variants.^15-17^ The cohort of 314 fibroblasts from patients with Mendelian disorders^17^ was used as controls.

### *In silico* analysis

*In silico* modelling of the CTR1 protein has been performed using PyMol Molecular Graphics System (v.2.4.1, Schrödinger, LLC) with AlphaFold model AF-O15431-F1 fitted to model 6M98.^18^ The CTR1 protein sequence (O15431) has been used as a BLAST query, and model organism sequences have been selected for comparison. Protein alignment has been performed using Clustal Omega (EMBL-EBI).

### Cell culture

Skin-derived fibroblasts of **P7** and controls were maintained in minimum essential medium (1 g/L glucose, 10% foetal calf serum and 1% penicillin–streptomycin) and grown to confluence in 75-cm^2^ flasks. When indicated, **P7** fibroblasts were treated with 50 μM or 100 μM copper histidinate (CuHis, Farmàcia Avel·lí Xalabarder Miramanda, Pujades, Barcelona, Spain) for 24 h or 72 h, respectively.

### Western blot

Fibroblasts were homogenized in RIPA lysis buffer containing protease and phosphatase inhibitors (Bio Basic Inc.). Electroblotting was performed in 4–15% gradient precast gels TGX^TM^ (Bio-Rad Laboratories, Hercules, CA, USA). Proteins were visualized by immunostaining with specific antibodies using the Odyssey LICOR fluorescent system. The antibodies used in this study were anti-SLC31A1 (ab129067, Abcam, Cambridge, UK) and anti-GAPDH (ab8245, Abcam, Cambridge, UK).

### High-resolution respirometry

High-resolution respirometry was performed using polarographic oxygen sensors in a two-chamber Oxygraph-2k system at 37°C according to the manufacturer’s instructions (Oroboros Instruments). Manual titration of OXPHOS inhibitors (Oligomycin, Antimycin) and uncouplers (CCCP) was performed using Hamilton syringes (Hamilton Company) as previously described.^19^ Data were recorded and analysed using the DatLab software v5.1.1.9 (Oroboros Instruments). Two biological replicates were performed for each experiment.

### Statistics and data analysis

Graphs and gene lists of proteins related to Cu metabolism or transport were analysed on the R Statistical Computing environment (https://www.r-project.org/). Statistical analyses of the biological assays were performed using one-way analysis of variance (ANOVA), followed by a post-hoc Bonferroni test to correct for multiple comparisons. Data were reported as the mean ± SD. *P*-values lower than 0.05 were considered statistically significant.

## Results

### Clinical findings

**Figure 1:**
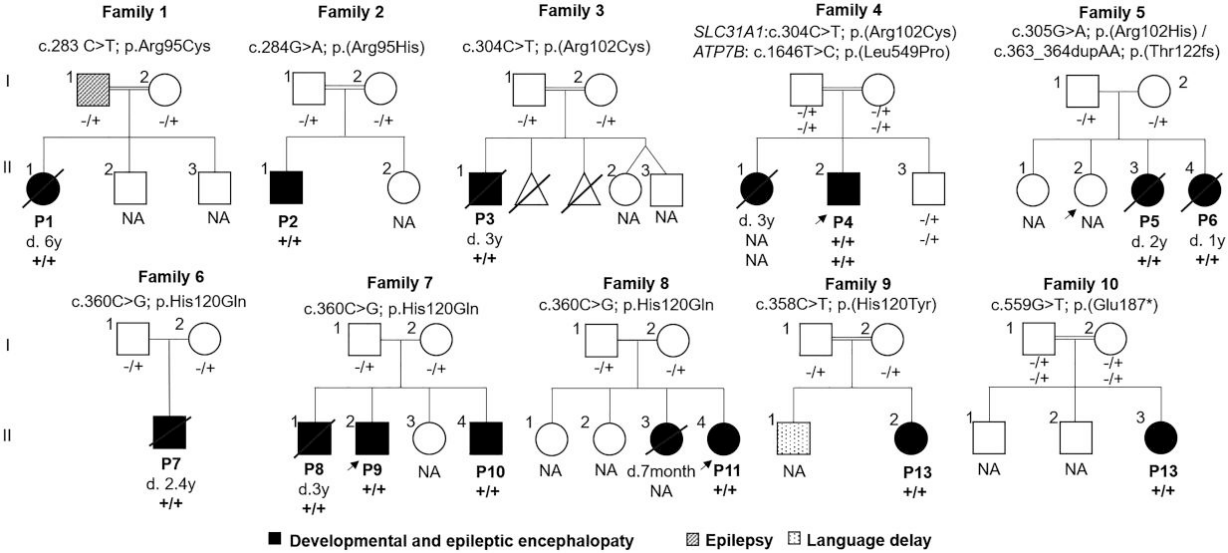
**Familiar pedigrees of the 13 cases included in the study (named P1 to 13).** Index cases are indicated with an arrow. NA: Not analysed. ‘+’ indicates the presence of the mutated allele, while ‘–’ represents the presence of the wild-type allele.

The clinical features, biochemical and genetic data from 13 cases (7 males/6 females) are summarized in Table 1. Further description of the clinical presentation of each subject (and human phenotype ontology descriptions) is included as Supplementary Materials Clinical Information and Supplementary Table 1 (patients identified as #P1 to P13). The pedigrees for seven cases are depicted in Fig. 1.

**Table 1:** Main clinical characteristics of the 13 cases described here

	This study, *N* = 13	%
Inheritance	11 Hom; 2 Comp Heter	84.6% Homoz
Variant^a^	7 missense, 2 Trunc.	77.7% Missense
Sex	6F:7M	
Death in infancy/childhood Age at last follow-up	7/13 3.7 (1–13) years	54
Clinical Features		
Severe global developmental delay	13/13	100
Hypotonia	12/12	100
Absent speech	12/12	100
Developmental regression	8/10	80
Epileptic encephalopathy	12/13	92
Microcephaly	7/9	78
Visual impairment	9/12	75
Dysphagia	8/11	73
Sensorineural hearing impairment	4/7	57
Failure to thrive	6/11	55%
Brain MRI		
Brain atrophy	11/12	92
Hyperintensity of cerebral white matter	9/11	82
Abnormal thalamic MRI signal intensity	8/11	73
Focal T2 hyperintense basal ganglia lesion	8/12	67
Abnormal brainstem MRI signal intensity	4/11	36
Subdural fluid collections	4/12	33
Biochemical anomalies		
Decreased copper	1/5	20
Decreased ceruloplasmin	1/5	20
Increased lactate	7/10	70

Further detailed information is included in Supplementary Table 1.

^a^Full variant description is in the text and Supplementary Table 2. Homoz, Homozygote; Comp Heter, Compound Heterozygote; Trunc, Truncating; F, Female; M, Male.

All patients presented with severe neurological impairment characterized by early-onset epileptic encephalopathy (EOEE), with an average age of onset of 6 months (range, 1–24 months). Intractable polymorphic seizures, described as myoclonic, tonic, clonic, and infantile spasms, were observed. Only three out of 13 patients had normal neurodevelopment prior to seizure onset. Physical examination revealed severe hypotonia without head control in all 13 cases, along with microcephaly in 7 out of 9. Additionally, movement disorders (tremor, parkinsonism, myoclonus, generalized chorea, and generalized dystonia) were present in seven of ten cases. Visual impairment (blindness, optic disc pallor, abnormal ocular movements, strabismus and cataracts) was noted in 9 out of 12 cases, and sensorineural hearing loss in 4 out of 7. As the disease progressed, most developed dysphagia (8/11), and one required ventilatory support. Seven cases died prematurely at an average age of 2.7 years (range, 1–6 years), mostly due to respiratory failure or epileptic decompensation.

Metabolic investigations revealed elevated lactate levels in plasma (*n* = 6/10) and CSF (*n* = 5/5) with increased pyruvate and alanine in one case. Serum copper and ceruloplasmin were normal in the four tested individuals, although one showed decreased circulating ceruloplasmin concentration.

**Figure 2:**
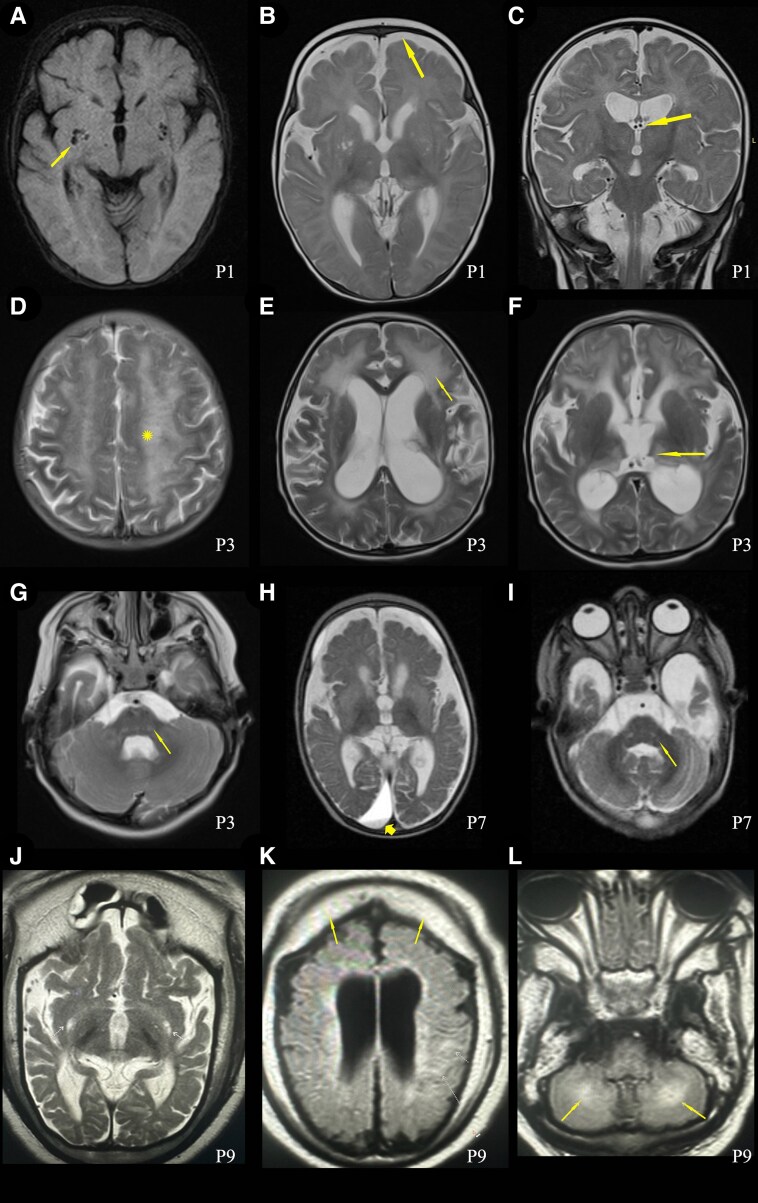
**Brain MRIs of cases P1 (A to C), P3 (D to G), P7 (H and I) and P9 (J to L). P1** at 6 months of age: (**A**) Prominent Virchow-Robin spaces can be seen in the lenticular nuclei and subganglionar area (axial, Fluid attenuated inversion recovery—FLAIR—T2). (**B**) Enlargement of the ventricular system and extraxial spaces without subdural fluid collections and corpus callosum thinning (axial, Fast spin echo—FSE—T2) (**C**) High T2 signal intensity in the pulvinar nuclei of the thalami (coronal, FSE T2). Myelination is delayed for a 6-month-old boy. **P3** at 3 years of age: (**D**) symmetrical hyperintensities in bilateral centrum semiovale with a tigroid pattern (axial, T2-weighted); (**E**) ventriculomegaly, hyperintensity in white matter periventricular and external capsule (T2-weighted axial), (**F**) loss of volume and abnormal signal in the thalamus (T2-weighted axial), (**G**) abnormal signal in the posterior pons and bilateral middle cerebellar peduncle (T2-weighted axial). **P7** at 9 months of age: (**H**) Hyperintense signal abnormalities in both the basal ganglia and diffuse brain atrophy prominently at bilateral frontotemporal lobes with secondary ventricular dilatation and frontal bilateral and right occipital subdural hygroma (T2-weighted axial). (**I**) Hyperintense signal abnormalities are observed in the bilateral brainstem, including severe temporal cortical-subcortical atrophy with significant enlargement of the subarachnoid space of the anterior temporal fossa. **P9** at 7 months of age; (**J, K**) severe frontotemporal cortical-subcortical atrophy with significant enlargement of the subarachnoid space of the bihemispheric convexity associated with frontoparietal subdural hygromas along with compensatory enlargement of the ventricular system and prominent thinning of the corpus callosum, (**L**) T2-WI hyperintense signal was observed throughout the upper left parietal lobe and medial cerebellar hemispheres (arrows).

Twelve patients received brain MRI, including three with follow-up imaging (Fig. 2, Table 1, and Supplementary Table 1). All affected individuals but one showed global brain atrophy, and compensatory enlargement of the ventricles in nine. Thinning of the corpus callosum or enlarged subarachnoid spaces was seen in six. Other common features included T2-WI hyperintensity involving the white matter, thalamus, basal ganglia, or brainstem. Radiological findings suggesting vigabatrin toxicity were observed in two cases. Intracranial haemorrhage was present in four individuals, involving the subarachnoid space, basal ganglia, or in the form of subdural haematomas. Other subdural fluid collections, such as hygromas, were observed in three cases.

### Genetic findings

The 13 cases presented here were found to have bi-allelic disease-causing variants in *SLC31A1*. We have identified eight different variants, of which seven were novel (Supplementary Tables 1 and 2). Nomenclature for each variant is determined using the canonical *SLC31A1* transcript NM_001859.4/ENST00000374212. Ten cases are homozygotes for missense variants p.Arg95His, p.(Arg95Cys), p.(Arg102Cys), p.His120Gln and p.(His120Tyr), one is homozygote for the premature stop codon p.(Glu187*) and two siblings are compound heterozygotes for the c.363_364dupAA; p.(Thr122LysfsTer8) and c.304C > T; p.(Arg102His) variants. The genetic position, *in silico* prediction scores, and American College of Medical Genetics and Genomics (ACMG) classification of the variants reported here, along with previously published variants, are summarized in Supplementary Table 2.

All the missense variants that affect highly conserved residues, conserved in all the assessed vertebrates (Supplementary Fig. 1), are absent or at a very low frequency in gnomAD (v. 4.1, accessed August 2024). We evaluated the gene–disease relationship (GDR) evidence following the Clinical Genome Resource (ClinGen) Clinical Validity Framework.^20^ Briefly, the strength of evidence for a GDR can be categorized as Definitive, Strong, Moderate, Limited, or No Known Disease Relationship. This is relevant because only genes with evidence of Moderate or above should be included in clinical diagnostic testing.^21^ For *SLC31A1,* we determined a Moderate level of evidence, confirming its association with autosomal recessive *SLC31A1*-related neurodevelopmental disorder.

**Figure 3:**
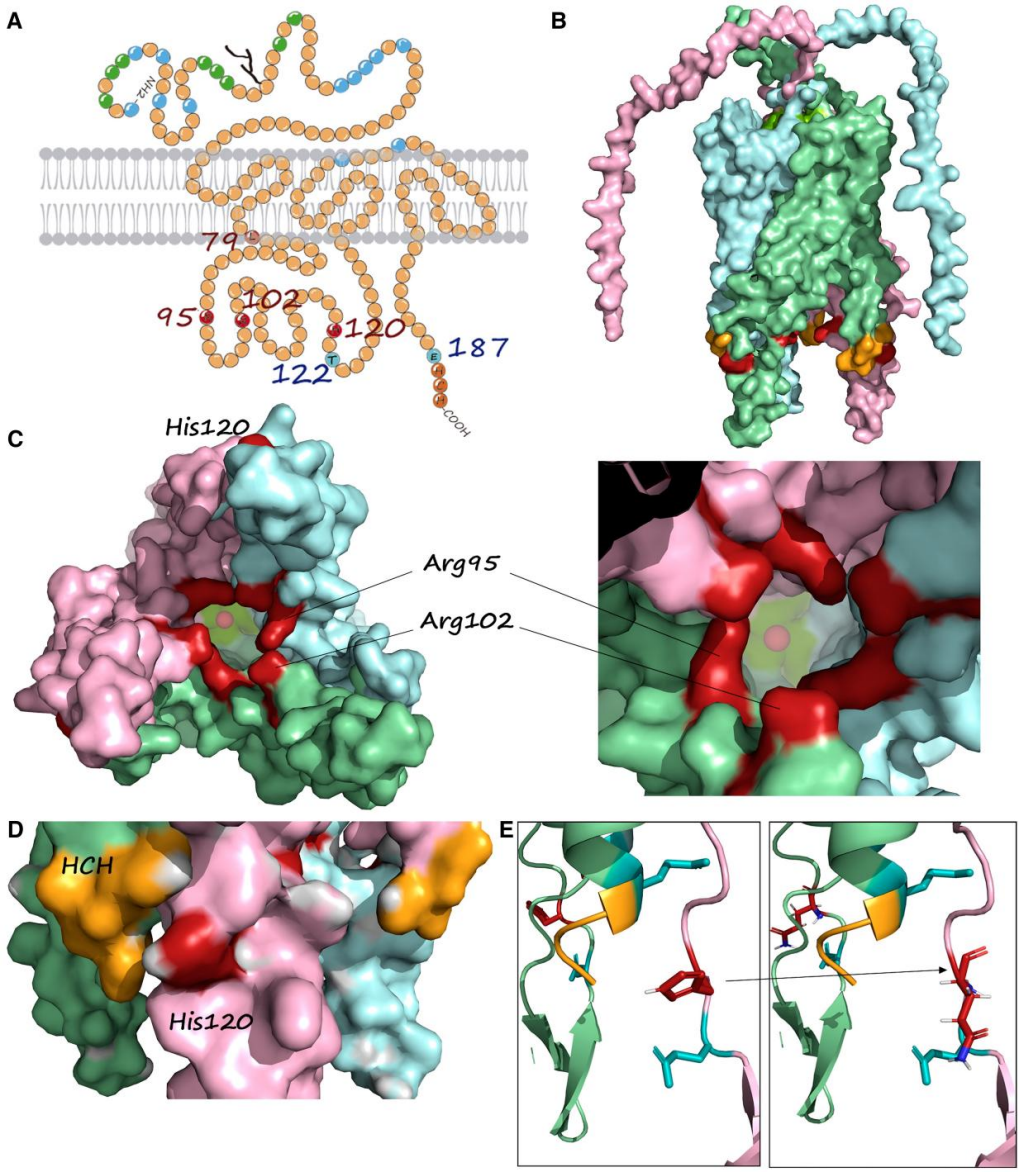
**CTR1 protein.** (**A**) Topological model of the human CTR1 protein. His and Met-rich domains are indicated in green and blue, respectively, and the HCH C-term domain is shown in orange. The residues Leu79, Arg95, Arg102, and His120, mutated in the SLC31A1 cases, are coloured magenta and the position of truncating variants at Thr122 and Glu187 are in violet. (**B**) Lateral view of the surface of the trimeric hCTR1, each monomer in a different colour. Mutated residues are shown in magenta, while the HCH domain is shown in orange. (**C**) Bottom (intracellular) view of the trimer showing Arg 95 and 102 at the ‘gate’ of the pore and His120 at the external surface. Copper is shown inside the pores as a red sphere. (**D**) Detailed lateral view showing His120 in close vicinity to HCH domain. **E**) Modelled substitution of His120 by Gln.

Variant c.283C > T (p.Arg95Cys), identified in **P1**, affects the same residue as the previously reported p.Arg95His also identified here in **P2**.^9^ This variant affects an alpha-helix in the cytoplasmic gate of the CTR1 channel (Fig. 3), close to the c.304C > T; p.(Arg102Cys) variant, identified here in two cases (**P3** and **P4**), while **P5** and **P6** are confirmed compound heterozygotes for the p.(Arg102His) variant that affects the same residue and the c.363_364dupAA variant that is predicted to skip the nonsense-mediated decay process and be translated into the C-terminus truncated protein p.(Thr122Lysfs*8). Five cases from three unrelated families (**P7-11**) bear the same transversion c.360C > G, which leads to a missense change of the electrically charged His to the non-charged hydrophilic Gln at residue 120 (p.His120Gln). The three families are of Hispanic origin (Spain and Mexico). The analysis of six variants in a 194 Kb region surrounding *SLC31A1* in cases P7 and P11shows that the variant is present on a common haplotype in the Mixed American population (11.7%), suggesting a potential founder effect (Supplementary Table 3). **P12** bears the c.358C > T change, affecting the same residue, p.(His120Tyr). His120 lies in the cytoplasmic region of the protein, in close vicinity to the metal-binding motif His-Cys-His domain at the C-terminal end of the protein (Fig. 3). Finally, **P13** has the c.559G > T variant, which results in predicted protein truncation at p.(Glu187*). This variant leads to the loss of the last three residues in the 190-residue protein, the CHC domain. Case **P4**, an individual of Egyptian origin, is homozygous for a missense variant in the *ATP7B* gene, which encodes a copper-transporting ATPase associated with Wilson disease (WD; MIM #277900). The variant identified, c.1646T > C; p.(Leu549Pro), has been previously reported in another Egyptian patient with WD^22^ and is classified as variant of unknown significance according to ACMG criteria (PM2, PP3). As functional studies were not conducted on this variant by Abdelghaffar *et al*.,^22^ and this region of ATP7B does not show significant constraint against missense variants (*Z* = −0.33, regional constraint according to gnomAD 4.1), its pathogenicity remains unclear, and the potential interaction between both aetiologies should be considered in the case of **P4**.

### Functional studies

RNA-seq was performed on **P7** fibroblasts and the *SLC31A1* expression levels were not significantly altered in comparison with the entire cohort of transcriptomes (Supplementary Fig. 2). Expression of genes related to Cu metabolism or transport that have been altered in other Cu-related pathologies was also within the normal range, including *ATOX1* and *CCS*, the cytoplasmic chaperones involved in Cu reception (Supplementary Figs. 3 and 4, expression data is reported in Supplementary Tables 4 and 5). Also, total CTR1 protein was assessed by Western blot in fibroblasts (**P7**) and lymphocytes (**P9**), both bearing the homozygous variant c.360C > G (p.His120Gln), and no differences were observed in these cell lines when compared with controls (Supplementary Fig. 5). Cu intracellular uptake was analysed in fibroblasts of **P7,** and no significant alterations were observed compared with controls studied in parallel (data not shown).

**Figure 4:**
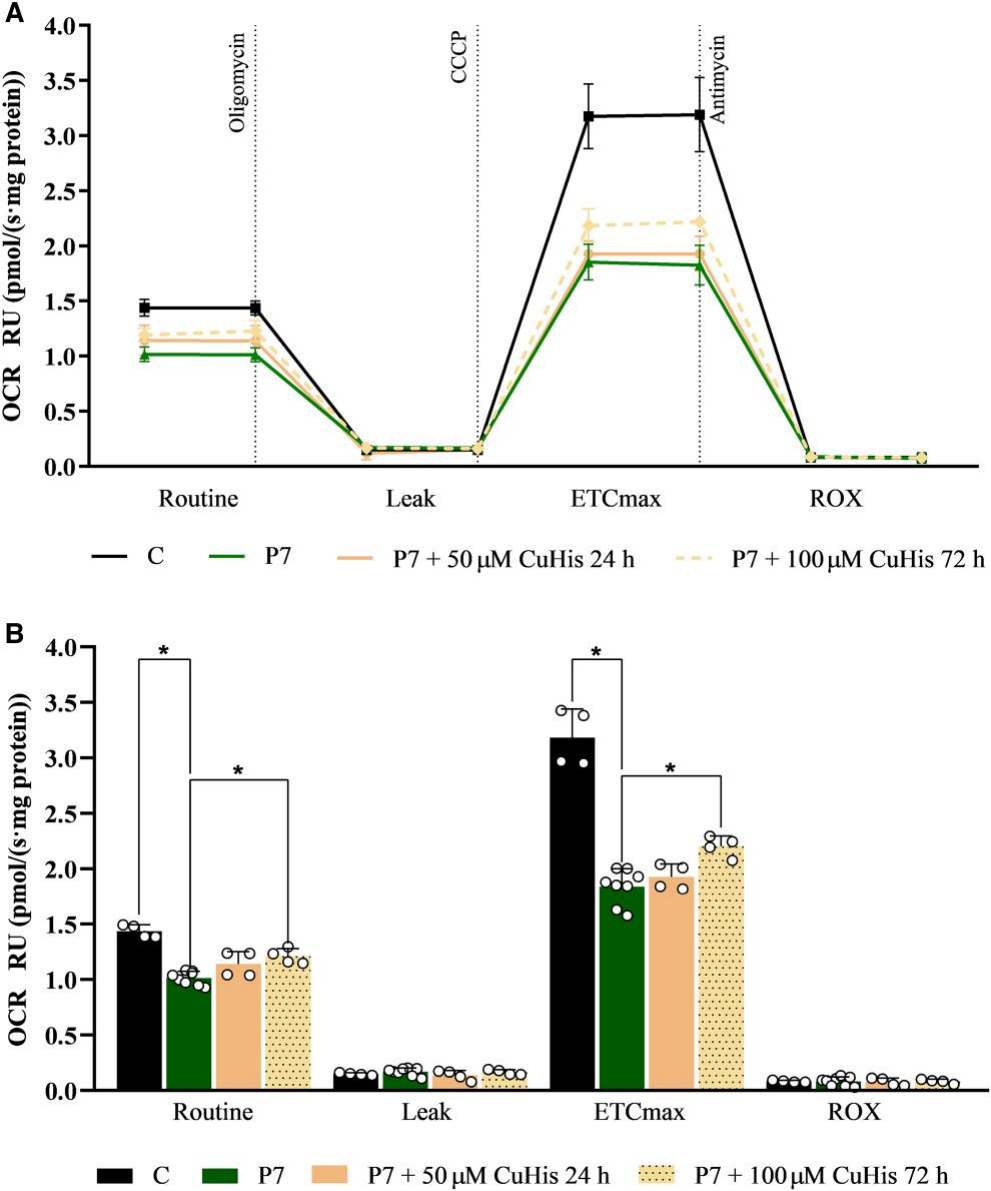
**Mitochondrial function assessment.** Mitochondrial respiratory capacity was analysed in P7 fibroblasts by investigating the oxygen consumption rate (OCR) with high-resolution respirometry. For each chamber of the respirometer, 2 million cells were used per condition (Control and P7 fibroblasts). OCR was measured as pmol/(second·mg of protein); therefore, all OCR values were normalized to milligrams of total protein. Data are expressed as relative units (RU) of non-treated P7 cells. (**A**) Scatter plot of mean OCR results across four time intervals delimited by the injection of the three compounds oligomycin, CCCP (carbonyl cyanide 3-chlorophenylhydrazone), and antimycin A. Dotted lines indicate the exposure of cells to each compound. For each of the four respiratory states, OCR values were measured in duplicate, allowing the calculation of mean values shown in the scatter plot. Two biological replicates were performed for each experiment. (**B**) Representation of mean results ± SD as a bar chart with statistical results (ANOVA: *F* (3, 16) = 29.51, *P* < 0.05; followed by a post-hoc Bonferroni test). White dots indicate single data-points of mean values (*N* = 4 for groups C, P7 + 50 μM CuHis 24 h, and P7 + 100 μM CuHis 72 h; and *N* = 8 for group P7) for the four respiratory states. A significant decrease in basal OCR was observed in P7 cells compared to the control (*, *P* < 0.05). P7 fibroblasts also exhibited a significant reduction in maximal respiratory capacity (*, *P* < 0.05) upon CCCP treatment. Treatment with 100 μM CuHis for 72 h, but not with 50 μM CuHis for 24 h, partially restored the mitochondrial respiratory capacity in P7 fibroblasts. Both basal and ETCmax respiratory rates were significantly increased (*, *P* < 0.05) upon 100 μM CuHis treatment (72 h) compared to non-treated P7 cells. C: control; CuHis, copper histidinate. OCR at basal state (ROUTINE); residual oxygen consumption after oligomycin treatment (LEAK); maximum oxygen consumption induced by CCCP titration (ETCmax); residual oxygen consumption after antimycin A treatment (ROX).

The mitochondrial respiratory capacity of **P7** fibroblasts was investigated by analysing the oxygen consumption rate (OCR) by high-resolution respirometry (Fig. 4 and Supplementary Table 6). Results showed an important impairment in **P7**’s fibroblasts’ basal respiratory rate compared to control cells (*P* < 0.05). In addition, P7 cells also had a significant reduction in their maximal respiratory capacity (*P* < 0.05) when treated with the mitochondrial uncoupler CCCP. Treatment of **P7** cells with 50 μM copper histidinate (CuHis) for 24 h did not show any significant respiration improvement. However, increasing the treatment dose up to 100 μM CuHis for 72 h resulted in a slight but significant improvement in respiratory capacity for the basal respiratory rate (*P* < 0.05) and for the maximal respiratory capacity (*P* < 0.05).

## Discussion

Here, we present the clinical and molecular findings of thirteen affected children with a recently described developmental encephalopathy caused by bi-allelic *SLC31A1* variants. The same homozygous variant (c.360C > G, p.His120Gln) was identified in three families. In most cases, the disease presented in the first year of life as a severe early-onset epileptic encephalopathy (EOEE) resulting in profound disability leading to early death before three years of age in half of the cases, supporting the high mortality risk associated with this clinical entity. Seven cases with recessive *SLC31A1* pathogenic variants have been reported in the literature.^9,10,23,24^ Comprehensive descriptions of the *SLC31A1*-related syndrome have been published in three individuals (Table 2)^9,10^ while phenotypic traits for four of them are not available.^23,24^

**Table 2:** Overview of the main characteristics of the 16 cases described so far

		This study	Batzios *et al.* 2022^9^	Dame *et al.* 2022^10^	All %	
Number of cases		13	2 sibs	1	16	
Variant (Protein)		Arg95Cys; Arg95His; Arg102Cys; Arg102His; His120Gln; Thr122fs; Glu187*	Arg95His	Leu79Pro		
Sex		6F:7M	NR	Male	6F:8M	
Age at last follow-up (years)		3.7 (1–13)	2	1 month	3.3	
Age at death (years)		2.7 (1–6)	Alive	0,1	2.4	
Death in infancy	HP:0001522	7/13	0/2	Yes	8/16	50
Clinical History						
Neurological features						
Severe global developmental delay	HP:0011344	13/13	2/2	Yes	16/16	100
Developmental regression	HP:0002376	8/10	2/2	No	10/13	77
Epileptic encephalopathy	HP:0200134	12/13	2/2	Yes	15/16	94
Age at seizures onset (months)		6 (1–24)	4	0.5	5.2	
Seizures	HP:0001250	12/12	2/2	Yes	15/15	100
Lethargy	HP:0001254	4/5	2/2	Yes	7/8	88
Hypotonia	HP:0001252	12/12	2/2	NR	14/14	100
Movement disorder	HP:0100022	7/10	0/2	No	7/13	54
Microcephaly	HP:0000252	7/9	0/2	No	7/12	58
Visual impairment	HP:0000505	9/12	2/2	NR	11/14	79
Sensorineural hearing impairment	HP:0000407	4/7	NR	NR	4/7	57
Absent speech	HP:0001344	12/12	2/2	Yes	15/15	100
Other clinical features						
Dysphagia	HP:0002015	8/11	2/2	NR	10/13	77
Failure to thrive	HP:0001508	6/11	2/2	NR	8/13	62
Brain MRI						
Global brain atrophy	HP:0002283	11/12	2/2	No	13/15	87
Cerebellar atrophy	HP:0001272	4/11	2/2	No	6/14	43
Ventriculomegaly	HP:0002119	9/11	2/2	No	11/14	79
Communicating hydrocephalus	HP:0001334	6/12	NR	NR	6/12	50
Noncommunicating hydrocephalus	HP:0010953	1/12	1/2	No	2/15	13
Intracranial haemorrhage	HP:0002170	4/12	0/2	Yes	5/15	33
Subdural hygromas		3/12	0/2	No	3/15	20
Subdural haematomas	HP:0100309	2/12	0/2	No	2/15	13%
Widened subarachnoid space	HP:0012704	6/12	0/2	No	6/15	40
Thin corpus callosum	HP:0033725	6/12	2/2	NR	8/14	57
Hyperintensity of cerebral white matter	HP:0030890	9/11	0/2	Yes	10/14	71
Focal T2 hyperintense basal ganglia lesion	HP:0007183	8/12	NR	NR	8/12	67
Abnormal thalamic MRI signal intensity	HP:0012696	8/11	NR	NR	8/11	73
Abnormal brainstem MRI signal intensity	HP:0012747	4/11	NR	NR	4/11	36
Tortuous cerebral arteries	HP:0004938	0/11	NR	Yes	1/12	8
Biochemical analyses						
Decreased circulating copper concentration	HP:0011967	1/5	0/2	Yes	2/8	25
Decreased CSF copper concentration	HP:0034823	NA	2/2	NA	2/2	100
Decreased circ. ceruloplasmin conc.	HP:0010837	1/5	0/2	Yes	2/8	25
Increased serum lactate	HP:0002151	6/10	2/2	Yes	9/13	69
Increased CSF lactate	HP:0002490	5/5	2/2	NA	7/7	100

NA, Not Assessed; NR, Not Reported (not clear if the trait is absent or NA or data not available); circ, ceruloplasmin; conc, circulating ceruloplasmin concentration.

The clinical presentation in the cases reported here closely resembles that of the twins documented by Batzios *et al*.^9^ (Table 2), who were homozygotes for the p.Arg95His variant, but contrasts with the congenital presentation observed in the case described by Dame *et al*.,^10^ who was homozygote for the p.(Leu79Pro) variant. The latter presented with major congenital anomalies involving the pulmonary, vascular, skeletal, and central nervous systems, seizures at 2 weeks of life, and acute intracranial haemorrhages diagnosed with cranial ultrasound. This proband appears to have the most severe presentation of this disease to date and is the only proband known to have low levels of serum copper and ceruloplasmin. Due to the retrospective nature of this report and the recent identification of this disorder, copper and ceruloplasmin levels were not assessed in most cases. However, these levels were measured in four out of five individuals harbouring the c.360C > G (p.His120Gln) variant, and all showed normal values. Case **P4**, who bears the p.(Arg102Cys) *SLC31A1* variant in addition to the p.(Leu549Pro) *ATP7B* variant, was identified to have low levels of serum copper and ceruloplasmin, together with low urine copper levels, the latter finding contrary to what would be expected in WD. Urine copper in other cases was not assessed, so it is unknown if SLC31A1 deficiency is responsible for his low copper urine excretion, and to which extent these two variants are altering the copper transport in this patient. **P12** is homozygote for the NM_000018.4:c.1246G > A p.(Ala416Thr) variant in the very long-chain acyl-CoA dehydrogenase gene (ACADVL *609575), which is associated with VLCAD deficiency (ACADVLD; MIM # 201475) (see supplementary clinical description). This particular variant has been previously described as associated with mild- or late-onset (adult form) disease, and functional analysis revealed that this variant maintains moderate activity.^25-28^ While this likely pathogenic variant may be modifying this proband’s phenotype to some extent, it does not seem responsible for the main clinical presentation. These two cases highlight the complexity in understanding and managing clinical presentations with dual diagnoses. Further functional studies would be necessary to fully understand the implication of each disease gene involved.

Biomarkers indicative of mitochondrial impairment were observed not only in our cohort but also in previously reported patients, reflecting the well-established role of copper (Cu) in mitochondrial metabolism. Notably, serum lactate was elevated in 60% of the analysed cases, and cerebrospinal fluid (CSF) lactate was elevated in all analysed cases, consistent with findings in previously reported cases.^9,10^ This mitochondrial dysfunction appears to significantly impact the clinical outcomes of the patients. Moreover, impaired mitochondrial respiratory chain activities in muscle and impaired mitochondrial respiration in fibroblasts were detected in the patient reported by Batzios *et al*.,^9^ and in **P7**, increased alanine in blood and CSF, elevated serum GDF-15 and FGF-21 and Krebs cycle metabolites in urine were detected, suggesting mitochondrial disease and leading to cofactors supplementation in this patient. Additionally, Leigh syndrome was suspected in two individuals who experienced developmental regression following infantile spasms, elevated lactate levels in both plasma and cerebrospinal fluid (CSF), and symmetrical T2-weighted hyperintensities in the basal ganglia, thalamus, and dentate nuclei. Therefore, defects in copper transporter receptors due to pathogenic variants in *SLC31A1* should be considered in the differential diagnosis of mitochondrial diseases.

The cases presented here display similarities with the clinical spectrum observed in other intracellular copper trafficking disorders, including rapid progressive neurodegeneration, intractable seizures, hypotonia, microcephaly, and severe brain atrophy since early infancy. These findings were frequently described in patients with Menkes disease,^29^ and Huppke–Brendel syndrome.^8^ Additionally, white matter hyperintensities in the bilateral centrum semiovale and subdural collections, such as hygromas and/or haematomas, are also described in Menkes disease.^29^ However, the subjects reported here did not show other typical neurocutaneous signs, including brittle hair, hypopigmentation, and connective tissue laxity as seen in Menkes, nor the ichthyosis and keratoderma observed in MEDNIK syndrome.^5^ Furthermore, there were no signs of tissue copper overload, such as hepatic cirrhosis, liver failure, or ocular Kayser–Fleisher rings, which are characteristic of Wilson disease.^30-33^

To date, experiments conducted on fibroblasts from patients with *SLC31A1* mutations (Batzios *et al.*^9^ and this study) have not revealed significant disruptions in CTR1 protein expression or localization. This suggests that, for most pathogenic variants, copper (Cu) impairment is likely not primarily due to cellular import. Instead, it is probably related to its delivery and trafficking within the cell, particularly to the mitochondrion (note that only total intracellular copper, not intramitochondrial copper, was measured). While further studies assessing mitochondrial copper levels, cytochrome oxidase activity and metal specificity would be valuable to clarify the precise link between copper metabolism and mitochondrial dysfunction, the limited availability of patient-derived fibroblasts has restricted our ability to pursue these analyses. Alternatively, other mechanisms not directly involving copper, where CTR1 cysteine residues play a key role, may be involved.^3^ Our study does not show an improvement in mitochondrial respiratory capacity in response to treatment with copper histidinate, unlike what was previously observed.^9^ It is unclear if this lack of response is due to methodological issues or to the particular variant and its effect on the CTR1 protein function. In this sense, it is interesting to notice the different positions of these mutations in the CTR1 channel. The CTR1 active channel is a transmembrane homotrimer (Fig. 3) that forms a pore that will import Cu from the extracellular space to the cytoplasm with high affinity.^34^ It has been proposed that four methionine residues (43, 45, 150 and 154) are involved in Cu transport through the core, and the cytoplasmic domain is involved in the controlled delivery of Cu to the corresponding chaperones (ATOX1, CCS, MT, etc.), which will distribute Cu intracellularly.^35^ All the variants identified in this study affect the first intracellular domain of CTR1 (residues 83-132; Fig. 3A). Residue Arg95 lies at the ‘gate’ of the internal cavity of the CTR1 pore (Fig. 3C),^18^ and its mutation for His has been proposed to alter the internal charges and Cu affinity.^9^ This residue is stabilized by H bonds with Arg91, and its substitution by His or Cys (like in the patients presented here) could impact both the intrapore electric charge and stability as well as the gate integrity. Previous studies showed that the substitution of His139 by Arg increased CTR1 *Km* values, demonstrating the relevance of the Arg and His equilibrium in the CTR1 Cu transport.^36,37^ The Arg102 also lies in the cytoplasmic domain, in close vicinity to Arg95 in the tridimensional CTR1 model (Fig. 3C). Again, the substitution of Arg by His or Cys seems to be disturbing the Cu transport through the pore and its delivery to the appropriate chaperones. His120 is also on the cytoplasmic loop of CTR1, but far from the gate. The 3D protein model locates this residue in close proximity to the HCH domain of the neighbouring monomer (Fig. 3D). This domain has been proven to be determinant in the delivery of Cu to the intracellular chaperones, especially ATOX1.^35^ In contrast, p.(Leu79Pro), described in the only patient presenting with low copper and ceruloplasmin circulating levels, introduces a proline residue in an alpha-helix of the first transmembrane domain, a change highly structurally disturbing, and probably affecting the pore shape and stability and thus completely blocking Cu import. The only patients bearing an early truncating variant (P5 and P6 sisters) were also bearing the p.(Arg102His) variant, and ceruloplasmin levels were not assessed (both passed away, and further functional studies are not available). To date, only 16 patients have been described, and clinical information remains limited, particularly regarding circulating copper and ceruloplasmin levels. Nevertheless, a genotype-phenotype correlation is emerging. Severe variants that disrupt the pore structure of the CTR1 channel appear to be associated with a more severe phenotype, characterized by low circulating copper and ceruloplasmin levels. In contrast, cytoplasmic mutations, potentially impacting intracellular copper trafficking, seem to result in a milder phenotype with copper and ceruloplasmin levels mostly within normal ranges. However, both forms are associated with mitochondrial dysfunction, epileptic encephalopathy, hypotonia, severe brain atrophy and death during early childhood. Notably, no loss-of-function homozygous cases have been identified so far, and no heterozygous cases with truncating variants leading to null alleles have been reported, suggesting that complete ablation of the SLC31A1 protein could be incompatible with life.

In summary, the comprehensive characterization of 13 patients with recessive *SLC31A1* variants, including the recurrent c.360C > G (p.His120Gln) variant, strengthens the association between CTR1 dysfunction and neurodegeneration in humans. The identification of shared clinical features and mitochondrial respiratory chain impairment provides valuable insights into the pathogenesis of this condition.

## Supplementary Material

fcaf348_Supplementary_Data

## Data Availability

The data that support the findings of this study are available from the corresponding author and patient caregivers upon reasonable request. Due to privacy and ethical restrictions, individual-level clinical and genomic data are not publicly available. However, all shareable gene expression data generated and analysed in this study, including reference codes for each individual, are provided in Supplementary Tables 4 and 5.
